# Supplementary material for: Are carbon emissions trading and green financial instruments synergistic? -Comprehensive quantitative research based on content analysis
Source: PLoS One. 2024 Mar 7;19(3):e0298601. doi: 10.1371/journal.pone.0298601 (PMC10919601; doi:10.1371/journal.pone.0298601)
Supplement: S1 Data — (ZIP) [file pone.0298601.s001.zip › Data/地级市Z_F工作报告.zip!/╣π╢1⁄2/╢1⁄2▌╕╩╨/└┤╫╘╣1⁄2╓┌║┼í╛┬φ┐╦╩2╛▌═°í┐_2018.docx]

各位代表:

现在,我代表市人民政府,向大会报告2017年政府工作,对2018年工作提出建议,请各位代表予以审议,并请市政协委员和其他列席人员提出意见。

2017年工作回顾

过去一年,在省委省政府和市委的正确领导下,市政府深入贯彻落实党的十八大、十八届历次全会精神,认真学习贯彻党的十九大精神,坚持以习近平总书记对广东工作重要批示精神为指导,按照省第十二次党代会的决策部署,紧紧围绕市第十四次党代会提出的“奋力在更高起点上实现更高水平发展,率先迈上基本实现社会主义现代化新征程”的要求,凝心聚力、真抓实干,积极运用八大调研成果,全面推进十大行动计划,着力抓好倍增计划、水污染治理攻坚战、城市品质三年提升计划、园区统筹组团发展、扶持次发达镇加快发展等一系列重点工作,较好地完成了市十六届人大一次会议确定的年度目标任务。

这一年,是东莞坚持稳中求进、经济运行提质提速的一年。预计全市实现生产总值7580亿元,同比增长8.2%左右,快于全国全省,为近四年来最高。进出口总额突破1.2万亿元。税收总额突破2000亿元。市场主体突破100万户。引进了历年投资规模最大的紫光芯云产业城等一批重大项目。新增上市企业数为历年之最。28个镇全部入选全国千强镇,13个镇入围前100名,虎门、长安进入500亿元俱乐部。村组两级集体经营总收入突破200亿元。全市经济稳中有进、进中向好的态势更趋明显。

这一年,是东莞聚焦“三个支撑”、发展动能显著增强的一年。供给侧结构性改革深入推进,规模以上工业增加值增速排名珠三角九市第二,先进制造业增加值占比突破50%,内资工业增加值、一般贸易进出口、高新技术产品出口占比均超四成。创新驱动发展能力不断增强,中国散裂中子源获得第一束中子束流,全市R&D投入占比升至全省第三,国家高企、省级创新科研团队、有效发明专利等数量稳居全省地级市首位,东莞成为全省唯一一个被纳入广深科技创新走廊的地级市。构建开放型经济新体制取得重大进展。滨海湾新区、东莞港正式挂牌,成为对接粤港澳大湾区的重要平台。在商务部首批向全国复制推广的24项试点经验中,东莞经验超过五分之一。在海关总署公布的中国外贸百强城市榜单中,东莞首次进入前三。

这一年,是东莞工作亮点纷呈、城市形象不断提升的一年。亚洲马拉松锦标赛、中国城市规划年会、两岸青年就业创业研讨会、中国音乐学院全国考级大赛总决赛等重大活动成功举办,加博会、海丝博览会、台博会、漫博会、智博会、高层次人才活动周、国际科技合作周科创会等重要展会取得丰硕成果。东莞三项工作在国务院大督查中获得表彰奖励,成功实现全国文明城市“四连冠”,首次获得综治工作最高荣誉“长安杯”,荣获“宽带中国”示范城市最佳实践奖,成为全国第九个版权示范城市、中国十佳会展城市,顺利通过国家节能减排财政政策综合示范城市和水生态文明城市考核验收。在中科院、腾讯等权威机构和企业发布的相关报告中,东莞综合经济竞争力、城市人口吸引力、智慧生活综合指数分别排名全国第14、第5和第6位。东莞充满活力的经济形象、生态宜居的城市形象、和谐友善的文明形象,得到进一步提升。

具体来说,我们突出抓好了以下工作:

一年来,我们以实施倍增计划为统领,着力扶持以先进制造业为核心的实体经济发展。大力推进重点企业规模与效益倍增计划。以市政府一号文出台意见,围绕政策、产业、土地等五大要素推出20条措施,选取214家市级、1054家镇级试点企业,从科技创新、发展总部经济、推进兼并重组、开展服务型制造、加强产业链整合、强化资本运作等六大路径发力,着力破解企业倍增发展的共性密码,“一企一策”解决企业个性难题。市级倍增企业主营业务收入超过6000亿元,增长27%以上;税收超过140亿元,增长29%以上,形成了一大批可复制推广的经验,得到国务院督导组和省的充分肯定。大力推进“实体经济十条”。落实省降低制造业成本若干政策,出台我市“实体经济十条”36项措施,全年为企业减负370亿元。全市规上工业企业实现每百元主营业务收入成本下降0.96元,利润总额大幅增长47.2%。加快打造智能制造全生态链。建成109条经济适用型示范线。长盈、华贝项目纳入国家智能制造新模式应用项目。“机器换人”专项资金申报项目2698个、总投资386亿元。全市机器人及智能装备制造企业163家,主营业务收入增长30%。扎实推动产业均衡发展。五大支柱产业有四个实现两位数增长。智能手机出货量3.56亿台,主营收入增长28%。华为、OPPO、VIVO手机出货量稳居全球前六。狠抓重大项目建设和招引。完成重大项目投资513.6亿元,超过计划19.6个百分点,增长16.9%,带动全市完成固定资产投资1682亿元,增长11.3%。新引进亿元以上内资项目175宗,协议金额1194.6亿元,增长41.9%;引进千万美元以上外资项目95宗,金额29亿美元,发展后劲进一步增强。

一年来,我们以广深科技创新走廊建设为契机,着力打造创新驱动发展升级版。积极优化创新环境。出台实施科技创新平台支撑计划等行动计划,发布广深科技创新走廊东莞段规划和中子科学城概念规划,启动广深高速创新资源带建设。大力培育创新主体。预计全市高企数量从2028家增至4077家,新增高企后备企业2400家,总量均居全省地级市第一。新增国家级孵化器培育单位11个、省创新科研团队5个、省创新创业领军人才3名。与北大、清华等38个知名高校和新型研发机构共建名校研究生院。东莞材料科学与技术省实验室进入全省首批启动的4个实验室行列。企业研发机构全年获省资助达6.2亿元,在全省地级市中大幅领先。落实研发费加计扣除政策,为1245家企业对应减免税款21.9亿元。推动科技金融融合。设立市级产业投资母基金,带动形成总规模超50亿元的“1+N”产业投资基金体系。镇街(园区)设立各类基金总规模超500亿元。备案登记基金增至387只,数量大幅增长87.9%。推动金融机构为科技企业发放贷款268.1亿元,增长37%。专利质押融资62.8亿元,占全省专利质押总额的58.2%。境内外上市企业、新三板挂牌企业分别增至43家和202家。企业通过IPO、增发、发债等累计融资116.5亿元,直接融资占比提高5个百分点,升至20%。金融对科技的支撑不断增强。

一年来,我们以构建开放型经济新体制综合试点试验为支撑,着力提升对外开放水平。全面深化综合试点试验。在全国率先实行“以企业为单元”的加工贸易监管模式等改革,形成了两批25项具有良好示范效应的改革做法。争取到国家在13个方面的政策支持。复制推广113项自贸试验区改革试点经验。口岸“三互”大通关实现全覆盖。国际贸易“单一窗口”建设扎实推进。省市共建深化商改综合试验基地揭牌。大力推动加工贸易创新发展。全市一般贸易(含保税物流)占比达46%。外企内销总额增长19%,占内外销比重达38.1%,提高3.1个百分点。加快培育新模式、新业态。“东莞制造+电子商务”深度融合。清溪保税物流中心(B型)、国际邮件互换局兼交换站投入运营。国际小包出口8821万件,增长24.3%,总量排全国第四。粤港跨境直通快线正式开通,实现粤港无缝清关,货物全程无障碍快速直达香港机场。东莞企业连锁经营门店超过3万家,景气指数全国第四。全市社会消费品零售总额2690亿元,增长10%。积极参与“一带一路”建设。开通俄罗斯进口班列,启动中欧双向班列。东莞始发国际班列货物贸易额增长42.1%。对“一带一路”国家及地区出口约1200亿元,增长10%。与美国威尔逊郡、利百伦市和剑桥市签订友好合作备忘录。东莞驻美国(旧金山)经贸办事处挂牌。

一年来,我们以推进水污染治理攻坚战为突破,着力提升生态文明建设水平。全面打响水污染治理攻坚战。立下“军令状”,以前所未有的力度,加快解决环保突出短板。新建截污管网767.8公里,新扩建9座污水处理厂,启动35座污水处理厂提标改造,铺开76条内河涌污染整治,扎实推进国考、省考断面水质保障工作。完成67家涉水污染企业整治和退出,工业废水排放总量削减167.2万吨。10条列入国家督办的黑臭水体,7条基本消除黑臭现象。河长制实现全覆盖。深入开展大气、固废等污染防治。完成2068家重点企业VOCs整治、391项扬尘污染治理,淘汰黄标车11877辆。全市空气质量达标天数301天。顺利达到国家“大气十条”终期考核要求。完成8座垃圾填埋场整治、3座环保热电厂技改增容,建成麻涌环保热电厂及餐厨垃圾处理厂工程,完成东南部卫生填埋场征地并启动建设工作。全市垃圾焚烧能力提升至11300吨/日,无害化处理率达100%。全面消除填埋场渗滤液直排。启动工业垃圾处理设施项目建设前期工作。加快修复城市生态功能。抓好林相改造和生态景观林带建设。建成麻涌新沙、道滘大罗沙湿地公园。东城、清溪、道滘被认定为省森林小镇。划定海洋生态红线,铁腕整治46宗历史遗留用海项目。完成30.7万亩永久基本农田划定工作。

一年来,我们以实施城市品质三年提升计划为载体,着力参与粤港澳大湾区建设发展。全面实施城市品质三年提升计划。聚焦“一心两廊三区四门户多节点”,围绕中心城区强化等10个重点领域,选定启动第一批次城市建设项目582个,投资规模达1880亿元。同步实施各镇街(园区)三年提升计划。新建美丽幸福村居100个。加快道路交通等基础设施建设。加强国铁、城际轨道项目前期研究,莞惠城轨建成通车,赣深客专东莞段开工建设。虎门高铁站、东莞西站、东莞火车站等TOD项目加快推进。从莞高速、粤晖大桥、红海大桥等项目完工通车。行政村光纤入户实现全覆盖,启动电网升级行动。提升城市精细化管理水平。出台城市精细化管理办法,查处城市“六乱”行为25万多宗,清理积存垃圾69.4万吨,增设公益广告牌近1.2万个。推进历史违建分类处理,拆除整改违建523宗、面积约61万平方米。在全省率先完成土规调整完善,土规、城规衔接率提至93%。启动建设用地使用权二级市场试点改革。农村土地承包经营权确权登记颁证率达93.8%,超额完成年度目标任务。新建农贸市场快检室150家、食品安全示范市场50家。市食品药品检测中心投入使用。积极融入粤港澳大湾区规划建设。规划8条地铁线路对接广州、深圳、惠州。扎实推进深莞惠3+2经济圈建设。签订深化穗莞战略合作框架协议,加强在规划、产业等八个领域合作。全力以赴做好全国文明城市迎检复评。深入实施“四大提升工程”和“十大专项行动”,大力推进85项重点任务。启动“九个一百”文明示范工作建设,建成文明示范项目930个。扎实推进友善之城、好人之城、志愿之城、希望之城建设。讲好东莞故事,传播城市魅力。

一年来,我们以实施园区统筹组团发展战略为抓手,着力提升区域协调发展水平。加大统筹发展力度。在不改变行政架构和空间范围的前提下,将全市划分为6大片区,谋划14个重点发展先行区,增强片区发展合力。松山湖片区“1+6”统筹组团发展试点取得突破性进展,松山湖在全国高新区的综合实力排名上升至23位。统筹滨海湾新区与东莞港建设,经省政府批准升格为省级开发区,设立管委会并将滨海湾新区面积扩容至83.2平方公里,成为粤港澳大湾区城市群发展规划重大发展平台。规划编制、项目填海、企业搬迁、理顺机制等工作加快推进。水乡经济区科学谋划新城与片区发展定位,启动水乡新城建设,片区生产总值增长9.3%,快于全市1.1个百分点。大力扶持次发达镇村发展。设立三年30亿元的专项资金,建立市直部门共同帮扶、重大事项议事协调等机制,推动次发达镇加快发展。8个次发达镇生产总值平均增长10.2%,快于全市2个百分点。70个次发达村(社区)村组两级经营纯收入增长14.5%,快于全市5.5个百分点。扎实推进对口帮扶和援建任务。开展东莞韶关对口帮扶,产业共建新签约项目117个、投资规模326.6亿元。推进韶关、揭阳精准扶贫精准脱贫工作,完成到村帮扶项目1317个,到户项目12.7万个,帮助20055人实现脱贫。落实东莞昭通“1+8”合作协议,在全国首创劳务协作扶贫援建模式。援藏援疆援川进展顺利,援建资金100%到位。探索合作共建新疆兵团41团草湖镇,扎实推进兵团草湖广东纺织服装产业园和西藏巴宜区小康示范镇村建设。与牡丹江市签订对口合作框架协议,全面完成“五个一”工作任务。

一年来,我们以强化民生保障为宗旨,着力推动社会和谐善治。狠抓维稳第一责任落实。以最高规格、最强部署、最严措施,全力保障十九大特别防护期的安全稳定。深入开展“飓风2017”专项行动,重拳打击“两抢”等突出违法犯罪,保持对黄赌毒违法犯罪严打高压态势,接报违法犯罪警情下降17.2%,刑事立案数下降14.3%。持续深入开展信访突出问题治理专项行动,妥善处置群体性事件和突发事件。社会大局保持和谐稳定。加强社会治理创新。深入推进“智网工程”建设,入格事项增至20大类83项,建成市镇村三级指挥调度平台。扎实推进“以案说防”和“一呼百应”工作。“平安建设促进会”实现镇街(园区)全覆盖。全面开展寄递物流、危化品、建筑施工、道路交通等专项整治,严厉打击“四黑”违法违规行为,大力推进消防安全“个十百千万”工程。全市排查整改各类安全隐患13.5万处,生产安全事故宗数、死亡人数、受伤人数分别下降17.3%、12.3%和29.4%,未发生重大及以上安全事故。积极推动文教体卫等社会事业发展。完成11所公办学校新建扩建工程。新增中小学(幼儿园)64所。新增随迁子女义务教育公办和补贴学位6.8万个,增长78.3%。民办义务教育优质学校比例达61.9%。东莞开放大学启用。东莞理工学院在全国大学综合实力排名提升47位,升至229位,其中理工类大学排名83位。顺利完成全国数字文化馆试点任务。公共文化服务标准化试点通过验收转为全国示范。成功举办市运会,获得全运会7个项目金牌,全民健身事业蓬勃发展。深入推进“医药分开”改革,为群众减轻医药费负担7600万元。选定5所医院建设区域中心医院。完善分级诊疗制度,推进家庭医生签约服务,启动基层医疗卫生服务能力建设三年提升工程。全面做好稳定房价、社会保障等其他工作。科学制定土地供应计划,多种方式供应住宅用地,构建保障房、人才房等住房体系,稳定房价初见成效。低保标准提高到每人每月880元。积极推进养老保险省级统筹和全民参保工作,40家医院接入国家异地就医联网平台。社会养老和医疗保险待遇水平稳步提升。发放就业创业补贴1.38亿元。成立关爱妇女儿童发展基金。慈善和养老事业、双拥创建不断加强。有效抵御“苗柏”、“天鸽”等灾害性天气。完成村级换届选举。统计工作进一步加强和改进。国防动员、工青妇幼、民族宗教、档案方志、防震减灾、残疾人、红十字会、打私人防等工作有效推进。

一年来,我们以提升行政服务效能为重点,着力强化与更高水平发展相适应的履职能力。深入学习贯彻党的十九大精神。深刻领会习近平新时代中国特色社会主义思想,切实强化“四个意识”和“四个自信”,把贯彻十九大精神和推动全市发展结合起来,全力以赴做好各项工作,真正做到学懂、弄通、做实。深化行政体制改革。组建成立市镇两级政务服务管理机构。公布首批1173个“最多跑一次”事项清单。“一门式一网式”改革实现市镇村三级联动。城市更新局正式挂牌。建筑市场全面放开。加强法治政府建设。出台市政府重大行政决策事项目录、合法性审查和后评估办法。完成《东莞市出租屋治安消防安全管理条例(草案)》起草工作。完成法治镇街和民主法治村(社区)年度创建任务,所有镇街达到省级法治创建标准。推动审计监督全覆盖。主动接受人大和政协监督,人大建议和政协提案办结率和满意率均达到100%。加强政府系统廉政教育,推进政府投资工程廉情预警评估系统建设。持之以恒纠正“四风”,工作作风进一步改进。

各位代表,2017年政府工作报告共分解工作任务355项,是近几年分解任务最多的一年。在各部门、各镇街(园区)的共同努力下,已完成及基本完成的任务343项,未完成的12项中,有7项是由于政策变动等客观原因导致。实际完成率为98.6%,基本兑现了对各位代表的承诺。对于未完成的任务,我们已责成相关单位形成书面材料在大会印发,向各位代表进行解释说明。今后,我们将进一步加大责任落实和工作推进力度,全力以赴完成各项目标任务。

各位代表,过去一年,全市上下众志成城,开拓进取,发展迈出了坚实步伐。这离不开上级和市委的正确领导,离不开历届党委政府打下的坚实基础,是全市人民共同奋斗的结果。在此,我代表东莞市人民政府,向全市干部群众,向人大代表和政协委员,向各民主党派、各人民团体、社会各界人士,向各驻莞单位、驻莞部队和武警官兵,向所有参与和支持东莞建设发展的港澳台同胞、海外侨胞和国际友人,表示衷心的感谢和崇高的敬意!

党的十九大明确指出:“中国特色社会主义进入新时代,我国社会主要矛盾已经转化为人民日益增长的美好生活需要和不平衡不充分的发展之间的矛盾”。十九大指出的社会主要矛盾变化在东莞也表现得比较明显,全市不少领域面临着发展不平衡不充分的矛盾问题,需要引起高度重视:在经济发展方面,主要是产业发展层次总体偏低,现代化经济体系不够健全。与国际接轨的经济管理体制和贸易方式尚未建立完善,吸引外资、稳定外贸增长的压力较大。同时,受空间限制和观念束缚导致工业投资、基建投资不理想。在创新驱动方面,新旧动能转换仍处于胶着状态。传统制造业智能化转型仍处于爬坡越坎阶段,新动能未能真正挑起大梁。虽然全社会已形成科技创新的共识,但持续创新并把创新转化成有效生产力的能力仍有待增强。在城市品质方面,主要是建设较为分散,未能做到“串珠成链”,经营城市理念不强,城市更新动力不足,交通拥堵问题日益突出,“两违”现象未能得到全面遏制,精细化管理水平仍需提升,与粤港澳大湾区重要节点城市的要求还有一定差距。在协调发展方面,区域发展不均衡情况依然存在,镇街发展差距比较明显。在生态环境方面,实现水环境的根本性改善仍需时日,大气、固废、土壤等污染防治任务艰巨。在社会治理方面,社会治理体系和治理能力与经济发展水平不相适应,矛盾纠纷仍然高发多发。在民生保障方面,优质教育资源供给不够丰富,文化、医疗、社保等公共服务的质量和均等化水平需要继续提升。在作风建设方面,一些干部懒政怠政和不作为、慢作为的现象仍然存在,攻坚克难锐气不够,一定程度上影响了工作推进。对于这些问题,我们必须有效加以解决。

2018年工作安排

去年十月,党的十九大胜利召开,为全国改革发展指明了前进的方向。今年是贯彻党的十九大精神的开局之年,是改革开放40周年,是决胜全面建成小康社会、实施“十三五”规划承上启下的关键一年,还是东莞升格地级市30周年,做好各项工作意义重大。省委要求,要推动十九大精神学习宣传贯彻往深里走、往实里抓,奋力把广东建设成为向世界展示习近平新时代中国特色社会主义思想的重要“窗口”和“示范区”。市委提出,要坚定习近平新时代中国特色社会主义思想引领,按照“新时代·新征程·新东莞”的总体要求,推动在创新转型发展、全面开放格局、区域协调发展、美丽东莞建设、社会和谐善治、文化繁荣兴盛上的“六大跃升”,奋力在更高起点上实现更高水平发展,努力在加快建设社会主义现代化新征程上走在前列。全市政府系统要按照这个总体要求,全力以赴抓好各项工作。

围绕“新时代·新征程·新东莞”的总体要求,我们必须准确把握发展大势,努力抓住机遇加快推动高质量发展。深刻领会中国特色社会主义进入了新时代这一新的历史方位,牢牢把握新时代东莞推动更高水平发展、实现“两个走在前列”的价值追求,紧紧抓住粤港澳大湾区建设、广深科技创新走廊建设等重大机遇,深入贯彻新发展理念,坚持质量第一、效益优先,进一步强化推进供给侧结构性改革、实施创新驱动发展战略、构建开放型经济新体制三大支撑,加快建设现代化经济体系,推动东莞实现更高质量、更有效率、更加公平、更可持续的发展。

围绕“新时代·新征程·新东莞”的总体要求,我们必须加快建设美丽东莞,努力打造优美环境、提升城市品质。积极适应后工业化时代城市发展的要求,以建设美丽东莞为目标,把生态环境治理和城市品质提升作为着力点和突破口,大力推进水污染治理攻坚战、魅力小城和美丽幸福村居建设、城市更新改造、精细化管理等重点工作,加快补齐环境短板,进一步夯实生态本底,全方位提升城市品质内涵,不断增强城市的综合承载力和区域竞争力。

围绕“新时代·新征程·新东莞”的总体要求,我们必须坚持以人民为中心,努力使全市人民获得感、幸福感、安全感更加充实、更有保障、更可持续。把执政为民作为所有工作的出发点和落脚点,着眼于人民群众对美好生活的向往,着力打造共建共治共享的社会治理格局,有效解决好群众关注的社会治安、食品安全、子女上学、看病养老等热点问题,量力而行、尽力而为推动教育、文化、医疗、社保等公共服务增量提质,加快推进人的全面发展、社会全面进步。

围绕“新时代·新征程·新东莞”的总体要求,我们必须加大改革创新力度,努力增创新的改革红利和发展动力。弘扬改革开放先行地的优良传统,坚决破除一切不合时宜的思想观念和体制机制弊端,突破利益固化的藩篱,积极推进中央和省赋予的各项改革试点任务,深入推进富有东莞特色的莞版改革,强化改革督察问效,力争在激发市场主体活力和创造力、发挥政府统筹发展作用、提升行政服务效率等方面取得新的突破,有效解决制约东莞发展的瓶颈问题,更好地向改革开放四十周年和东莞升格地级市三十周年献礼。

围绕“新时代·新征程·新东莞”的总体要求,我们必须狠抓工作执行落实,努力将上级和市委的决策部署转化为具体行动和实际成效。坚决听从以习近平同志为核心的党中央指挥,集中精力聚焦做实,排出任务书、时间表、路线图,全力以赴完成好上级和市委部署的任务,把各项决策和要求具体化为工作项目、转化为落实举措,一件接着一件办,一年接着一年干,一步一步推动全市宏伟蓝图变为美好现实,久久为功,善作善成。

今年政府工作的指导思想是:全面贯彻党的十九大和中央经济工作会议精神,坚持以习近平新时代中国特色社会主义思想为指引,按照省委十二届三次全会和市委十四届五次全会的部署,坚持稳中求进工作总基调,牢牢把握高质量发展的根本要求,大力发展实体经济、强化创新驱动、扩大改革开放、提升城市品质、建设美丽东莞、增进民生福祉,加快解决发展不平衡不充分的问题,奋力推动东莞在更高起点上实现更高水平发展,努力在全面建成小康社会、加快建设社会主义现代化新征程上走在前列。

全市经济社会发展的主要预期目标为:生产总值增长8%;人均生产总值增长8%左右;固定资产投资总额增长15%;社会消费品零售总额增长9%;进出口总额增长3.5%;市一般公共预算收入增长9.5%;R&D投入占比提高至2.6%;国家高企数量达4500家;先进制造业、高技术制造业增加值占比分别达到51%和42%;一般贸易(含保税物流)占比达48%;新建截污管网1300公里;城镇生活污水处理率达到95%以上;空气质量达标天数310天以上;PM2.5浓度控制在35微克/立方米以内;全面完成年度节能减排任务;新提供随迁子女义务教育公办和补贴学位13万个;城乡居民人均可支配收入增长8.5%以上。围绕上述目标和要求,各级各部门必须全面落实上级和市委各项决策部署,突出抓好以下工作:

一、把发展经济的着力点放在实体经济上,加快打造粤港澳大湾区先进制造业中心

深入实施倍增计划。将市级试点企业动态扩容至250家以上。加强对镇级试点企业指导,总结推广先进经验。全面落实20条干货措施和22项配套政策,用好专业服务资源池、产权补办、并购重组基金等一揽子创新举措,以倍增扶持拓宽企业成长空间。调整完善资金拨付、督查督导和综合评价机制。探索将成熟的定向扶持政策扩展至全市企业,推动广大企业加快实现全域倍增。

积极构建智能制造全生态链。加快推进自动化、智能化改造,培育300个以上改造应用项目,建设2-3个智能制造示范项目。培育一批企业智能升级系统解决方案服务商。强化智能制造要素支撑,筹建国家智能制造装备监督检验中心,实施万名智能制造人才提升工程,力争3年内撬动融资支持100亿元以上。发展机器人智能装备产业,认定15-20个市首台(套)重点技术装备产品。加快建设工业互联网。

着力培植更多优势支柱产业集群。实施智能终端产业生态系统建设计划,加快推进华为、OPPO、VIVO、紫光等重大项目建设,补强核心芯片、高端显示屏等关键配套,着力巩固电子信息产业的优势和地位。推动传统产业优化升级,进一步做强纺织服装、食品饮料、家具制造等产业集群。扶持新材料、机器人、人工智能、生物医药、第三代半导体、现代建筑等产业加快发展,谋划大科学装置关联前沿产业布局,着力培植和打造新的优势支柱产业集群。

以产品质量为突破口推动“质量东莞”、“品牌东莞”建设。大力推进质量强市建设,实施技术标准战略和消费品“三同”工程,开展区域品牌打造行动,推动智能终端、毛织等创建质量安全示范区。抓好商标品牌培育、提升、保护和服务四大工程,加强“东莞制造·知名商标”认定和管理。加强顶层设计,构建领先全国、对接国际的技能人才培养体系,打造“技能人才之都”。继续办好加博会、台博会、漫博会和智博会等展会,建立会展全球合作联盟,推动“东莞制造全球行”,不断提升东莞制造的知名度和美誉度。

打造更加有利于实体经济发展的环境。深入推进“三去一降一补”,突出“破”“立”“降”,推动企业成为供给侧结构性改革主力军。全面落实省市“实体经济十条”,统筹用好30亿元扶持企业专项资金。在去年减负新高的基础上,力争年内再为实体经济减负190亿元以上。灵活确定工业用地出让年限,鼓励“先租后让”使用土地,划定365平方公里工业保护线。规范反映突出的中介服务。探索制订新型产业用地政策体系。深入推进解决重点制造业企业用地历史遗留问题试点工作。抓好建设用地使用权二级市场试点改革。做好第四次全国经济普查。弘扬企业家精神和工匠精神,鼓励更多社会主体投身创新创业。

二、积极参与广深科技创新走廊建设,加快向创新型一线城市挺进

深入实施创新驱动发展升级版行动计划。启动高企“树标提质”工程。“一业一策”培育重点领域高新技术产业。实施核心技术攻关“攀登计划”。建设东莞国际技术转移中心,认定一批海外创新中心,构建国际科技合作网络。制定推动科技成果转化的政策。加大对企业研发财政补助力度,力争规上企业研发机构建有率达38%。推进知识产权综合管理试点改革,为创建国家知识产权强市打下基础。积极申报省高新技术产业开发区。

大力抓好广深高速创新带“1+1+11”示范建设。成立专项小组,按照定规划、定项目、定标准、定政策的要求,制定总体建设方案,引进培育一批重大项目,启动实施一批环境提升和城市更新项目,进一步提升沿线环境和科技平台建设水平。出台政策,支持沿线镇街(园区)在科技创新、经营城市、盘活土地、利益共享、协调发展等方面先行先试,探索经验,引领带动东莞全域创新发展。

抓好松山湖、中子科学城等重大创新平台建设。深入推进松山湖片区“1+6”统筹组团发展工作,抓好16个重点先行区建设和原东部工业园企石片区开发,集聚一批重点实验室和工程技术研究中心。抓好中子科学城规划建设,围绕建设综合性国家科学中心的目标,加快完成规划设计、土地整备和基础设施建设,引入同步辐射光源等大科学装置,尽快形成区域创新共同体。推动东莞材料科学与技术省实验室建设,确定运营模式和建设方案,加快集聚一批国际化、高水平的研发机构和龙头企业,打造具有重大国际影响的先进材料创新高地。

大力促进科技金融产业融合发展。用好4年2.5亿元的发展利用资本市场专项资金,积极推动企业上市,力争境内外上市、新三板挂牌企业总数达到260家。抓好众创金融街、龙湾梧桐小镇、松山湖基金小镇、东城金融产业集聚区等建设,争取已备案私募基金突破400只。培育发展创业投资、风险投资、融资租赁等新兴业态。鼓励国企积极参与科技金融产业融合发展。完善地方金融监管体制,强化对涉众型金融风险的防控,维护金融稳定。

三、增创改革开放先行地新红利,加快培育国际经济合作和竞争新优势

进一步激发改革活力。以开放型经济新体制综合试点试验为契机,大力推进贸易自由化便利化改革等34项重点改革事项,力争形成20项以上东莞特色改革经验。深化园区统筹组团发展改革,总结松山湖片区试点经验,探索在水乡、滨海等片区全面推开。深化商事制度改革,抓好省市共建深化商改综合试验基地建设,铺开“双随机一公开”改革,打造事中事后监管全国样本。实施乡村振兴战略,推进全国农村集体产权制度改革试点工作,促进农村集体经济与实业投资、金融信托等国企合作,拓展投资渠道。完成农村土地承包经营权确权登记颁证工作。抓好城市管理和综合执法体制、盐业体制和供销社综合改革。强化改革正向激励,建立健全改革容错纠错机制,进一步调动各级锐意改革、勇于创新的积极性。

以滨海湾新区为龙头加快融入粤港澳大湾区建设。高标准、高起点推进滨海湾新区建设,抓好新区规划编制、基础建设、招商引资和东莞港全域规划、港口资源整合等工作,加快引进聚集一批高端创新资源。坚持“规划引领、项目带动、机制保障”,进一步推动滨海片区组团发展。扎实推进综合保税区的建设和运营。加快水乡新城建设,优化管委会职能和机构设置,加强产业招商统筹,加速平安金融科技城等项目建设,着力打造粤港澳大湾区国际商务港。深化穗莞战略合作,突出创新走廊共建、港口航运合作、交通互联互通、生态环境联治,加强高端服务业特别是高端医疗、教育和人才等方面的合作,推进“穗莞政务跨城通办”试点。积极参与深莞惠经济圈“3+2”建设。主动对接广东自贸区。加强与港澳台在科技、金融、商贸、公共服务等领域的合作。

着力促进内外源经济协调发展。探索建立科学有效的加工贸易创新发展综合评价体系,打造一批示范企业、园区和项目,培育一批具有特色的国际商品交易平台,发展一批跨境电商、保税物流、现代供应链等贸易新业态、新模式,推动加工贸易向全球价值链高端跃升。深入实施“亲企清政”工程,推进万名民营企业家培训计划,继续开展大型骨干企业、成长型中小企业培育行动,引导社会资本加快进入以先进制造业为核心的实体经济。全面推进市属国企重组整合,力争在交通运营、城市更新、金融产业、科技创新、水环境治理、能源投资等领域重组整合一批具有核心竞争力的龙头骨干集团。

积极拓展“一带一路”合作空间。办好海丝博览会,进一步提升展会的国际化、专业化和市场化水平。加快中外运石龙码头改扩建等工程建设,推动跨境班列双向对开常态运作,积极申报多式联运监管中心。加强与内蒙古、新疆等沿边地区的口岸通关协作。举办“迈向新丝路”走出去系列活动。力争新增企业境外投资项目超过100宗。

四、加大招商引资和有效投资力度,全面夯实更高水平发展的后劲

掀起主动出击引进大型优质项目的新热潮。整合全市招商资源,加强专业招商队伍建设,出台新的招商引资奖励政策,探索产业基金招商新模式。提高招商谋划水平,加强产业发展前瞻性、支撑性研究,坚持先进制造业、总部性平台、加速器营运平台、以自持物业为主的平台开发商等“四个优先”,着力引进一批龙头企业和核心项目。加强国际经贸合作网点和国内产业合作联络处建设,深化与知名产业基金、创投基金等的合作,积极开展合作招商。探索建立健全全市及片区重大项目招商引资统筹和流转、利益分享等机制,对重大项目实行“一事一议”。力争年内引进超亿元内资项目110宗、超千万美元外资项目100宗。

千方百计为项目落地拓展空间。从园区平台土地整合、闲置用地处置和“三旧”改造等方面入手,进一步盘活存量土地利用空间,优先保障重大项目和重大平台用地需求。对工业用地或仓储用地在符合规划和不改变土地用途的前提下,调整容积率等规划指标不再增收土地价款差额。出台硬措施,未来三年盘活存量土地3万亩,整合1000亩以上连片土地20块。

想方设法推动有效投资增长。出台进一步促进有效投资增长的实施意见。建立投资项目调度管理联席会议制度。按照“储备一批、开工一批、投产一批”的要求,建立2018-2020年滚动投资项目库,对项目建设实行全流程、规范化管理。制定财政投资建设项目前期工作办法及配套政策,精简投资审批流程,提高服务效率。严格执行企业投资项目准入负面清单制度,下放投资项目审批权限。落实以备案制为主的企业投资准入管理体制。大幅提高年度考核中投资增长、项目建设的分值和权重,根据完成情况分别进行奖励和约谈。建立投资项目数据库与投资统计台账,确保应统尽统。

不遗余力抓好重大项目建设。修订重大项目管理办法,突出项目产业和效益导向,实施全链条管理。加大重大项目土地、资金、人才等资源保障力度,简化规划、用地、报建等审批服务程序,实行重大项目“马上就办”。完善市领导挂钩督导、督查巡查、现场协调等推进机制,切实加快项目建设。探索建立重大产业项目后评价机制,提高项目监管和履约水平。力争年内完成重大项目投资600亿元,增长15%以上,推动66个项目新开工、43个项目投产。

五、深入实施城市品质三年提升计划,不断提高城市承载力与竞争力

探索建立与城市建设相适应的投融资体制。树立经营城市理念,深化城市建设投融资研究,加强对土地储备的协调领导和规划统筹,摸清存量建设用地情况,做好TOD等重点领域的土地储备及整合工作。深化在库储备土地城市规划,提升土地价值。创新投融资机制,推广运用PPP等模式,更好地推进地铁1号线、环莞三期等项目建设。用好总规模200亿元的莞信基础设施和公共服务投资基金,通过股权投资带动项目融资,保障重大项目资金需求。鼓励镇街(园区)组建基础设施投资基金。推动东实集团、水投集团、交投集团加快资产证券化步伐,提升融资能力,促进轨道交通等重大项目建设。鼓励村集体参与重大基础设施建设。充分发挥市场机制作用,吸引莞民投、民盈集团等民间资本参与经营性项目建设与运营。

加快构建新“三位一体”的城市中心体系。加强中心城区、松山湖、滨海湾新区“三心”之间的交通、市政等基础设施网络联系,实现城市功能有机疏散、错位发展。适度提升中心城区首位度,抓好“两轴三节点”城市品质提升,加快南城国际商务区规划建设,启动环同沙地区规划建设及整治提升,推进“三江六岸”统筹开发。加快建设魅力小城,出台设计技术指引,每个镇街(园区)选取至少1条道路和1个片区进行示范提升。打造美丽幸福村居,启动第一批3个市级示范片区的申报建设工作,探索实施镇级连片建设。开展城市生态修复、城市功能修补,用好山水资源,进一步彰显“一半山水一半城,城市就在山水间”的城市特色。

狠抓城市品质三年提升计划项目实施。做好项目统筹谋划,出台项目管理、财政激励、建设指引等政策,优化审批流程,落实工作责任,全面掀起项目建设高潮。按照“完成一批、启动一批、谋划一批”的要求,大力推进400多个、总投资超1700亿元的项目建设,每个镇街打造建成2-3个示范项目,让群众尽快感受到城市品质提升带来的明显变化。

全力推进城市更新和TOD开发。抓紧修编城市更新专项规划,出台城市更新规划单元划定标准及管理办法,制定集体经济组织公开选择合作企业的操作指引、连片改造专项政策,组建城市更新基金,鼓励“工改工”连片开发。重点推进南城东华、麻涌滨江、樟木头樟洋、凤岗天堂围、长安科技商务区等总面积1.7万亩的8个片区改造。支持常平国际创新港、黄江灵狮小镇等4个旧工业区活化更新。推进挂影洲围统筹开发。加快东莞火车站、东莞东火车站、常平火车站、虎门高铁站、东莞西站、松山湖北站、谢岗银瓶站以及深茂铁路滨海湾站等的TOD开发,划定首期轨道站点TID项目用地范围,探索轨道交通站点车辆段上盖物业开发的可行性,提前介入、策划新线车站地下空间,提高轨道交通综合经营能力。

抓好地铁1号线等基础设施建设。全面推进赣深客专东莞段建设,完成深茂铁路东莞段前期工作,加快穗莞深、佛莞城际东莞段工程建设。抓好穗莞深轨道衔接工作,适时启动寮步高铁始发主站规划,构建与湾区中心、区域重大枢纽快速联系。开工建设地铁1号线一期工程,争取完成2号线三期项目立项,加快推进虎门二桥、莞番高速、深圳外环高速东莞段等建设,对水乡大道等实施快速化改造,对与深广惠交接的20个国省县道门户路段进行改造提升,打通一批断头路,进一步完善市域综合交通一张网。实施电网升级行动,力争完成投资30.6亿元。建设光网无线城市,新增4G基站1万个、光缆6500公里,打造全省一流、全国领先的信息基础设施。

大力整治交通拥堵、“两违”等突出问题。按照“建、控、活、优、增、投、管、减、合”的思路,进一步强化交通拥堵整治统筹领导,出台系统科学、标本兼治的整治方案,力争一年实现拥堵状况得到有效缓解,三年实现交通通行能力明显提升。抓紧改造环城路等7个拥堵节点,实施道路微循环改造。升级交通指挥系统。编制公交专用道网络规划,加大对常规公交投入。加强出租车、网约车、共享单车管理。新建22座人行天桥,完善城市慢行系统。开展历史遗留违法建筑普查摸底,建立信息数据库和台账。探索实施分类处理,逐步消化存量违法建筑。严厉打击新增违法建筑,对违规建设销售小产权房从源头进行精准打击。强化“两违”整治联合执法力度,每月通报整改和拆除情况,对不力的镇街进行约谈、问责。

努力让城市更有序、更干净、更靓丽。充分发挥“智网工程”在城市网格化、精细化管理中的重要作用,进一步加强信息系统、指挥调度体系、网格管理队伍等建设,推动更多的部门入格,提升基层化解问题的能力和水平。推广东坑经验,推进城市“六乱”、生活噪音等综合整治。打造精品工程,提升东莞大道、中心广场、黄旗山城市公园等的景观和品质,加强高铁站、火车站、汽车站和旅游景点等区域管理,每个镇街打造至少1个上档次的城市公园。加强公厕规划、管理和建设。实施“填坑补绿”行动,抓好道路设施、园林绿化等改造提升,全面消除道路坑洼、人行道塌陷、绿化管养不到位等现象。开展城市精细化管理考核,逐步实现精细化管理全覆盖。进一步丰富城市建设的文化内涵,努力让城市更有品位。

六、加快创建国家生态文明建设示范市,满足人民日益增长的优美生态环境需要

深入推进水污染治理攻坚战。年内新建截污管网1300公里,争取1600公里。抓好破损截污主干管网修复,健全管网维护保养机制。同步实施接水通水工程,加快推动厂网一体化运营,充分发挥截污管网作用。改扩建污水处理厂6家,提标改造35家,加快推进首批分散式污水处理设施建设,将全市污水处理能力提升至356.5万立方米/日。加大对企业排污的监管力度,从严处理偷排等违法行为。实施考核断面水质保障工程,深入推进“八大专项行动、十项整改措施”。2018年春节前完成40条重污染河涌整治示范项目,年内完成不少于100条内河涌整治。

加强固废、大气、土壤等治理。建成东南部卫生填埋场、市区有机资源再生利用工程等项目,启动工业垃圾处理设施项目建设,加快厚街环保热电厂技改增容工程,推进建筑垃圾和余泥渣土消纳场建设,力争年内在全省率先实现新增生活垃圾全焚烧、零填埋。出台生活垃圾强制分类工作方案。抓好垃圾填埋场渗滤液的全面收集处理。加大力度推进存量垃圾综合治理,逐步整治39座污染重、库容大的填埋场。完成124个砂场(含洗砂场)的整治。统筹推进大气污染防治行动计划,重点整治臭氧、PM2.5等造成的污染,完成印刷、塑胶等重点行业VOCs污染整治任务。加强施工扬尘污染治理,有效整治泥头车撒漏现象。深入推进“土十条”。开展土壤污染详查,实施重点监管企业名录制度,完成石碣、麻涌、洪梅土壤修复试点工程。

大力抓好生态系统保护。加强“两区一园”保护建设工作。加快推进东莞植物园二期、银瓶山三期项目建设,完成大屏嶂、大岭山森林公园和同沙生态公园品质提升前期工作。启动黄江巍峨山、谢岗银山、企石东丫湖等公园建设。创建国家生态园林城市和省森林小镇。加强地质灾害隐患点治理和海岸线修复整治。完成15个易涝点整治,做好黄沙河流域海绵城市试点工作。建设海洋环境在线监测系统。

发展绿色、低碳、循环经济。实施能效倍增行动,推动500家用能单位5年内实现能效倍增。积极推动装配式建筑,规模化发展绿色建筑。保持好农用地的生产与生态功能,稳定粮食生产能力,强化粮食安全保障,做大做强高端精品农业。积极建设国家生态文明先行示范区,加快创建国家生态文明示范市。

七、着力改善民生和创新社会治理,不断提升人民获得感、幸福感和安全感

优先发展教育事业。扩充公共教育资源,新建扩建公办中小学20所。开展小区配套幼儿园专项整治。力争公办幼儿园占比未达30%的镇街(园区)新建(新增)1所以上公办幼儿园。努力挖掘公办学位潜力,增加民办学位补贴数量,向随迁子女新提供学位13万个。创建品牌学校30所,推进教育集团化办学。制定民办教育“1+N”政策文件,进一步提升民办学校教育质量。实施“互联网+教育”战略,新增优课微课1万节。抓好高中阶段学校考试招生制度省级试点改革。改革职业教育人才培养模式,积极创建省现代职业教育综合改革示范市。建设高水平理工科大学。深化名校研究生联合培养工作。大力推动老年教育发展。不断提升全民教育水平。

加强社会保障体系建设。进一步完善养老保险和医保制度,将中央、省属机关事业单位纳入机关养老保险,建立医保按病种分值付费方式,推广工伤预防试点经验,推进社保医疗O2O服务平台建设,做好基本养老金调整工作。实施积极的就业创业政策,全面放开小额创业贷款户籍条件,促进高校毕业生和就业困难人员等重点群体稳定就业。出台构建科学住房体系的配套政策,搭建住房租赁监管平台,加强人才住房保障工作,抓好房地产调控、去库存工作,扩大住房公积金制度覆盖面,助推房地产市场健康平稳发展。完善社会救助体系,启动社会综合福利院建设,鼓励和支持社会力量参与养老事业。出台新的拥军优属实施办法,深化军民融合,做好全国双拥模范城中期考核迎检。

扎实做好市内外帮扶工作。大力推进次发达镇加快发展,制定资金池竞争性分配办法,落实共同帮扶机制,推动引进更多高质量项目,力争4个以上次发达镇生产总值突破100亿元。大力推动次发达村(社区)加快发展,基本实现次发达村(社区)村组两级经营性纯收入稳定在300万元以上或比2015年增长20%。抓好东莞韶关对口帮扶工作,加快华南装备园、莞韶园、县级共建园区的建设和招商。做好对韶关、揭阳精准扶贫精准脱贫工作,确保贫困村内100%相对贫困户脱贫、100%相对贫困村出列。扎实推进新疆兵团草湖工业园和41团草湖镇共建工作。加快西藏巴宜区小康示范镇村建设。做好东莞昭通扶贫协作工作。深入开展与牡丹江市对口合作。加强与四川雅江县、九龙县和重庆巫山县的对口联系。

推进“健康东莞”建设。全面深化医药卫生体制改革。优化整合医疗资源,加快5所区域中心医院建设,推进医疗联合体建设,推动部分公立医院向专科医院、医养结合机构等转型,完成150所社区卫生机构的升级达标。推进公立医院药品跨区域联合集中采购改革。建成市儿童医院。启动国医馆、市中西医结合医院建设,打造中医药强市。建立符合医疗卫生行业特点的薪酬制度。全面推进家庭医生签约服务。成立市医疗和健康保障中心。举办1000场健康讲座。每个镇街选择1-2个以上农贸市场进行提升改造。启动国家食品安全示范城市创建。

健全公共安全防控体系。严厉打击“两抢”犯罪,始终保持对涉枪、涉管制刀具、涉金融和“黄赌毒黑拐骗”等违法犯罪的高压态势,力争各类刑事犯罪立案数下降8%以上,其中“两抢”警情数、立案数分别下降30%和40%以上。出台加强公安工作意见。抓好流动人口和出租屋基础信息采集。推进辅警队伍正规化建设。探索建立警区警务统筹联动工作机制。推进社会治安视频监控系统建设。启用新情报指挥中心。推广铁骑勤务。抓好危险化学品安全综合治理,加强安全生产标准化建设,落实企业全员安全生产责任制。健全出租屋、三小场所、高层建筑消防安全长效治理机制,建设消防安全“技防”系统,推进电气火灾综合治理,挂牌督办一批火灾隐患。提高应对自然灾害及其引发的次生、衍生灾害的能力。完善应急管理体制机制,强化突发事件处置组织领导,提升基层预防和处置水平。

推动文化体育事业繁荣发展。巩固全国文明城市“四连冠”成果,培育和践行社会主义核心价值观,广泛开展理想信念教育,加强公民道德建设,启动“新东莞文明美丽乡村”建设,打造一批“友善企业”、“文明社区”,推动文明创建全域化、长效化。大力实施文化惠民工程,开展全民艺术普及行动计划,加快图书馆、文化馆总分馆制建设,推动基层综合性文化服务中心全覆盖。落实上级部署,筹划组织纪念改革开放40周年系列文化活动,创作一批优秀文艺作品。做好红色革命遗址保护利用工作,加强文物保护利用和文化遗产传承。加快筹建市博物馆新馆。积极申报国家历史文化名城。打造虎门“中国近代史开篇地”文化旅游品牌。优化体育公共服务体系建设,开放50个公共体育设施,开展万人体育公益培训。办好第一届市民运动会。迎战省运会。抓好男篮世界杯筹备工作。推动档案数字化,推进市档案中心和新方志馆建设。

八、提振与新时代·新征程·新东莞相适应的精气神,建设人民满意的服务型政府

打造一体化的政务服务体系。加强政务服务改革顶层设计。深入推进公共服务事项标准化,按“最小颗粒度”的要求细化办事指南和流程,让企业和群众办事更加便捷。探索推进相对集中行政许可权,实施部门行政审批职能整合归并。按照全市一盘棋的要求,统筹全市政务信息化建设,促进政务信息资源共享,加快“数字政府”改革和一体化政务服务平台建设。加强政府网站建设与管理,全面推进政务服务公开,努力使政务服务工作走在全省前列。

全面推进依法行政。加强改进政府立法工作,将一批成熟的经验和做法以规范性文件、政府规章、地方法规的形式固化下来。完善政府法律顾问制度,深入推进行政复议应诉试点工作,加大培训力度,不断提高政府工作人员法治思维和依法行政能力。以依法及时解决群众合理诉求为核心,全面构建新时代信访工作机制体制,着力解决反映强烈、社会关注度高的信访问题。抓好“七五”普法。建成市镇村三级公共法律服务实体平台网。力争90%以上村(社区)达到省级民主法治村(社区)创建标准。

切实加强廉政建设。严格落实中央八项规定精神,进一步纠正“四风”问题。依法接受人大及其常委会的监督,自觉接受政协的民主监督,接受社会和舆论监督。坚定不移惩治腐败,认真查找廉政风险点,完善审计改革配套制度,完善政府投资工程廉情预警评估系统,大力整治腐败问题,增强群众认同感。

进一步强化主动作为的责任意识和担当意识。深入推进“三大建设”,教育和引导各级领导干部牢牢把握正确的政治方向,进一步增强“四个意识”和“四个自信”。正确处理好勇于担当、依法办事、干成事业之间的关系,增强狠抓落实的本领,提振攻坚克难的锐气,以更高的要求、更优的作风、更实的举措,高质高效地完成全年各项目标任务。

各位代表!潮平两岸阔,风正一帆悬。让我们乘着改革开放四十周年的春风,高举习近平新时代中国特色社会主义思想伟大旗帜,在省委、省政府和市委的坚强领导下,与全市人民一道,锐意进取,主动作为,真抓实干,共同谱写更高水平发展新篇章,再创东莞现代化建设新辉煌!
